# Assessment of the association between plant-based dietary exposures and cardiovascular disease risk profile in sub-Saharan Africa: a systematic review

**DOI:** 10.1186/s12889-022-12724-w

**Published:** 2022-02-19

**Authors:** Tatum Lopes, Annalise E. Zemlin, Rajiv T. Erasmus, Samukelisiwe S. Madlala, Mieke Faber, Andre P. Kengne

**Affiliations:** 1 Non-Communicable Diseases Research Unit, South African Medical Research Council, PO Box 19070, Tygerberg, Cape Town, 7505 South Africa; 2 Division of Chemical Pathology, Department of Pathology, Faculty of Medicine and Health Sciences, University of Stellenbosch, Tygerberg Hospital, Cape Town, South Africa; 3 Division of Chemical Pathology, Department of Pathology, Faculty of Medicine and Health Sciences, University of Stellenbosch, and National Health Laboratory Service (NHLS), Tygerberg Hospital, Cape Town, South Africa; 4 School of Public Health, University of the Western Cape, Bellville, South Africa; 5 Department of Medicine, University of Cape Town, Cape Town, South Africa

**Keywords:** Plant-based, Dietary exposure, CVD risk profile, Africa, Systematic review

## Abstract

**Background:**

Studies have investigated dietary attributes associated with cardiovascular disease (CVD) risk in Africa. However, there has been no effort to critically assess the existing evidence. This systematic review examined available evidence on the association between plant-based dietary exposures and CVD risk profile in Africa. PROSPERO registration number: CRD42020159862.

**Methods:**

We conducted a literature search for observational studies reporting on plant-based dietary exposures in relation to CVD risk profile in African populations. PubMed-Medline, Scopus, EBSCOhost, and African Journals Online platforms were searched up to 19 March 2021. Titles and abstracts of the identified records were screened independently by two investigators. The quality of the studies was also assessed independently.

**Results:**

Of 458 entries identified, 15 studies published between 2002 and 2020 were included in this review. These studies originated from 12 sub-Saharan Africa (SSA) countries. Sample sizes ranged from 110 to 2362, age from 18 to 80 years; and majority of participants were females (66.0%). In all, four plant-based dietary exposures were identified across SSA. Sixty percent of the studies reported a significant association between a plant-based dietary exposure with at least one CVD risk factor such as hypertension, diabetes mellitus, dyslipidaemia, overweight/obesity, and metabolic syndrome.

**Conclusions:**

The few available studies suggest that there may be a protective effect of plant-based dietary exposures on CVD risk profile in the African setting. Nonetheless, more elaborated studies are still needed to address plant-based diet (PBD) adherence in relation with CVD risk in African populations.

**Supplementary Information:**

The online version contains supplementary material available at 10.1186/s12889-022-12724-w.

## Background

In 2019, the World Health Organization (WHO) Global Health Estimates reported that non-communicable disease (NCD) was responsible for almost three quarters (74%) of the global mortality. Furthermore, of these NCDs, two cardiovascular diseases (CVD) namely ischaemic heart disease and stroke were the top two causes and contributed to 16 and 11% of the number of global deaths, respectively [1]. A similar trend was observed in sub-Saharan Africa (SSA), where ischaemic heart disease (39%) and stroke (34%) were responsible for most of the CVD related deaths that were reported in 2017 [2]. The burden of NCDs has increased to such an extent that it now surpasses that of infectious diseases globally and within the African region. CVD accounts for 57% of NCD deaths in Africa, translating into nearly 2 million deaths in 2016 [3]. Although the mortality rate of NCDs in SSA (35%), is slightly lower than the continental and global statistics it should not be underestimated [2]. In 2019, it was also reported that 463 million people worldwide had diabetes mellitus (DM), which ranked ninth in the WHO’s top ten causes of global deaths. In terms of the world regional prevalence, Africa has about 3.9% (19.4 million) of its adult population living with DM [4]. In SSA, DM is prevalent in 3.5% of the population and still largely undiagnosed (≥ 40% of cases) [2], however, it may also contribute to the rise in NCDs.

The nutrition transition [5] is believed to have driven most of the epidemiological change pertaining to the physical activity levels and dietary habits of populations worldwide. Stage one of the transition dates to the era of hunter gatherers (approx. 12,000 years ago) [6]; who consumed a diet with a low content of fat and a high content of fibre and carbohydrates. The second stage was dominated by the famine, which was represented by low levels of food availability and diversity. The famine that affected SSA, occurred towards the end of the twentieth century and continued up until the early twenty-first century [7]. During stage three (the receding famine): there was a reduced intake of carbohydrates, increased consumption of fruits, vegetables, and protein. Stage four is associated with a dietary shift that included a low-fibre diet with high contents of fat, cholesterol, sugar, and refined carbohydrates (i.e., processed and ultra-processed foods), which are thought to have been consumed by African populations as early as the 1980s [8]. This latter stage of the nutrition transition, was consequently accompanied by a high prevalence of obesity in SSA, which has mainly increased since the 1990s and 2010s, based on data from national surveys [8]. The fifth and current stage of the nutrition transition for most countries: compromises a diet that is rich in fruits, vegetables, and complex carbohydrates, with a reduced intake of animal foods namely dairy products and meat, also limiting processed foods and fat. Stage five is suggested to be the health-conscious phase that brings about a change in the dietary behaviour of individuals [9].

A shift in the demographic and epidemiological profiles of populations occurs concurrently with the nutrition transition. Together these transitions have partially accelerated the development of NCDs in Africa and other low-to-middle income countries [10]. Rapid advances in the global trade and industry sector of many African countries; in tandem with the increased migration from the rural to urban settings, and adopting Western cultures further contribute to the current burden of nutrition related NCDs. Food systems across the world and in SSA [8] have been modernized, whereby, minimally processed traditional diets are increasingly replaced with highly processed foods that have a high fat and sugar content that are typical of a Western diet. To counter these adverse effects of the nutrition transition, it has been recommended that dietary patterns become more plant-based [11] to sustain the health of populations and the global food systems [12].

Since the early 2000s, the term “plant-based” has gained popularity in the field of nutritional epidemiology [13], and of late it has also been endorsed in the clinical settings of some high income countries [14–18]. Several variations of this term exists: “whole-food plant-based diet” [14], “plant-based diet” [11, 15–25], “plant-based dietary patterns” [26, 27] and “plant-based dietary practices” [28]. As a result, there is still some uncertainty and a lack of consensus regarding the definition of a plant-based diet (PBD) [29]. This disparity could thus delay the recommendation of PBDs as part of the prevention and control strategies in low-to-middle income countries with a high burden of NCDs. Nonetheless, according to the literature a PBD is primarily said to encourage the consumption of healthy plant foods and discourage the consumption of less healthy processed plant and animal foods [29].

There is a growing literature on the cardioprotective effects of consuming plant foods, and it has consequently been recommended as an adjunct therapy for chronic diseases and in some cases an alternative to drug therapy and surgical procedures [14–16, 18]. In addition to this, plant-based dietary indices have been developed [23] and plant-based dietary patterns [26, 29] are increasingly being identified and assessed regarding the associations between PBD adherence and health outcomes. The evidence-based literature on the benefits of PBDs in the prevention and management of NCDs, such as DM [24] and CVDs [21, 22] is, however, predominantly documented in high income countries. This research area is still largely under addressed in low-to-middle income countries in Africa; therefore, this review aimed to assess the association between plant-based dietary exposures with CVD risk in African populations. This systematic review addressed the following questions: 1) What is the association between plant-based dietary exposures and CVD risk profile in African populations? 2) How consistently has a PBD been assessed across studies investigating its association with CVD risk profile in African populations?

## Methods

This systematic review was conducted according to the principles of the Preferred Reporting Items for Systematic Reviews and Meta-Analyses (PRISMA), and findings are reported in keeping with its recently published 2020 statement [30] and checklist (Additional file 1).

### Study selection

The Population, Exposure, Comparator for the exposure and desired Outcome (PECO) concept was applied to retrieve studies [31]. The review only focused on quantitative observational studies (i.e., cross-sectional, cohort and case-control), that examined the association between plant-based dietary exposures and CVD risk in African populations. Studies that were conducted in adults aged 18 years or older, and who were residing in SSA met the inclusion criteria. The dietary exposure was defined as the consumption of at least one or more primary components of a PBD i.e., fruits, vegetables, whole grains, legumes, nuts, and seeds. Studies were included if their dietary exposure was plant-based either assessing healthy plant foods, nutrients derived from plants and/or plant-based dietary patterns [29]. Additionally, studies that investigated a comparator dietary exposure, if any, that was reminiscent to the Western diet [32], or contained processed plant or animal foods that are characteristic of unhealthy dietary patterns were eligible. The dietary comparator included any unhealthy dietary patterns, which was defined as a diet that mainly contains animal foods such as processed or red meat, eggs, and high-fat dairy products or less healthy plant foods e.g., sweets and desserts [25]. CVD risk factors were the health outcomes that were assessed: hypertension, dysglycaemia and DM, dyslipidaemia, overweight/obesity, and metabolic syndrome. Hypertension was defined as a blood pressure measurement of 140/90 mmHg [33], DM as fasting and 2-h glucose values ≥7.0 and 11.0 mmol/L, respectively [34], dyslipidaemia and metabolic syndrome was not defined, but acceptable if it was informed by country and/or gender-specific management guidelines, and overweight/obesity was defined using the WHO classifications. However, we did not exclude studies if they applied different CVD risk factor definitions, guidelines and/or reference intervals. Studies were excluded if: the study design was not observational, the study did not report measures of association, was conducted in non-African populations, assessed health outcomes other than CVD risk, the article was not written in English or French which are the predominant official languages in SSA, and if they were performed in children (younger than 18 years) or non-human subjects. Articles published prior to 1990 were not included, because very few to no studies investigated “plant-based” intake before the 2000s [13], and research publications on CVD risk factors only emerged from SSA during the late 1990s [2].

### Literature search

We searched the literature for published studies that were conducted in African populations between January 1990 and March 2021. PubMed-Medline, Scopus, EBSCOhost databases and the African Journals Online (AJOL) platform were last searched on the 9th, 10th, 17th, and 19th of March 2021, respectively. The search terms were adapted for each of the databases as previously reported for PubMed-Medline in the protocol of this systematic review [35] and are further outlined in the supplementary material (Additional file 2). The AJOL platform was searched to identify studies that were published in local journals. In addition to this, our search strategy consisted of the African filter [36] with relevant free texts and/or medical subject headings. Our search strategy also applied filters/limits for the publication year, age of study participants, and language restrictions (i.e., articles written in English and French), where this was deemed to be necessary or possible (Additional file 2). Grey literature was searched up to the 19th of April 2021 for relevant conference proceedings using the Web of Science citation index.

### Screening

The EndNote X8 citation management software was utilized for records retrieved up to March 2021. The latter application was used to identify duplicates prior to screening the search results. Two investigators (TL and SSM) independently screened the titles and abstracts (TIABs) of the articles that were identified. A third investigator was consulted during the full text assessment when discrepancies could not be resolved. Manual searches were conducted by screening the reference lists of relevant studies to identify other articles of interest.

### Data extraction and synthesis

Data was captured and managed using the Excel Workbooks for systematic reviews (VonVille, Helena M. Primary Excel Workbook for Systematic Reviews, http://libguides.sph.uth.tmc.edu/excel_SR_workbook). A list of the non-eligible studies was documented, and the exclusion reasons were recorded in an Excel Workbook (Additional file 3). Data extraction was performed by TL and checked by SSM. The following data items were extracted from the individual studies; the first author’s name, year of publication, country name, study design, sample size, characteristics of the study population, dietary exposure, and comparator (if any), reported measures of CVD risk outcome (e.g., blood pressure measurements, and/or biochemical tests), and measures of association between plant-based dietary exposures and CVD risk such as odds ratios (ORs). Findings are reported overall and by country, study design, study setting and population (rural versus urban), dietary exposure, health outcome assessed and significant findings.

### Quality assessment

Studies included were examined by two investigators (TL and SSM) to assess their risk of bias. This was performed independently using the National Heart, Lung, and Blood Institute (NHLBI) Quality Assessment Tool for Observational studies [37]. The quality scores ranged from 0 to 14 depending on the study design. The methodological quality of each study was classified as good (score above 11), fair (score between 6 and 9), or poor (score below 6) [38].

### Registration and protocol

The protocol for this systematic review was registered on the International Prospective Register of Systematic Reviews (PROSPERO), registration number CRD42020159862, and is available online [35].

## Results

### Study selection

**Fig. 1:**
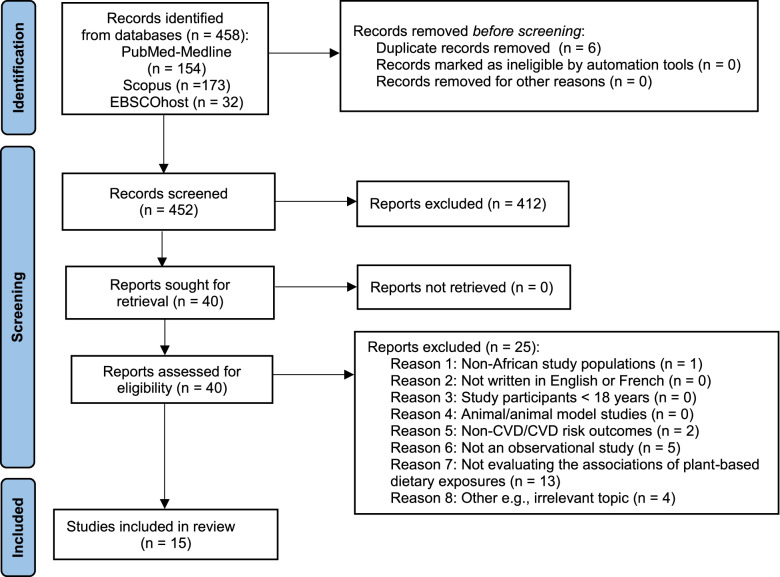
PRISMA Flow diagram of the study selection

Our literature search yielded 458 records: 154 were identified through PubMed-Medline, 173 from the Scopus databases, 32 via EBSCOhost and 99 through the AJOL platforms. No additional publications on the association between PBDs and CVD risk in Africa, apart from this systematic review’s protocol [35] was identified via the Conference Proceedings Citation Index (CPCI). Six duplicates were identified prior to screening the TIABs. During the TIABs screening we identified two articles that reported findings from the same study cohort, and the most comprehensive publication was selected. Four hundred and twelve studies were excluded based on the reasons summarized in Fig. 1. After full text assessment, fifteen studies met the inclusion criteria. Nine of the eligible studies were retrieved from PubMed-Medline [39–50], and three from the EBSCOhost platform [51–53]. No additional studies were identified after manually screening the reference lists of the included studies.

### Study characteristics

Included studies originated from 12 countries across SSA: (*n* = 1) Kenya [44], (*n* = 1) Botswana [52], (*n =* 1) Zambia [42], (*n =* 1) Ghana [48], (*n =* 1) Democratic Republic of the Congo (DRC) [50], (*n =* 1) Ethiopia [53], (*n* = 3) Tanzania [43, 45, 47], and (*n* = 7) South Africa [39–41, 46, 47, 49, 51]. One study [47] had four recruitment sites namely Nigeria, Tanzania, South Africa, and Uganda. In addition to this, five African countries namely Mozambique, Nigeria, South Africa, Sudan, and Uganda participated in a multi-country study [39]. According to the World Bank data [54], 2 of the 12 SSA countries are classified as upper middle income [40, 41, 46, 49, 51, 52], 6 are low-to-middle income [42–45, 47, 48, 50], and 4 are low-income countries [39, 47, 53]. The included studies (*n* = 15) were published from the year 2002 to 2020. There was low heterogeneity across studies in terms of study design: 13 were cross-sectional [40–48, 50–53], 1 cohort [49] and 1 case-control [39]. Eighty-seven percent of the included studies had a cross-sectional study design with comparable health outcomes (e.g., 7 of the 15 studies assessed overweight/obesity). However, the included studies reported on different dietary exposures, association measures, and were conducted in study populations with different characteristics (Tables 1 and 2) that could not be pooled to conduct meta-analyses. Therefore, we provide a narrative summary of their findings. The total number of participants across included studies was 11,088, and per study, the number of participants ranged from 110 to 2362. All participants were adults 18 years or older, and the proportion of men ranged from 0 to 52%. Two of the studies did not provide clear estimates for the age of their participants and three studies did not assess a dietary comparator; therefore, we did not extract data for these variables (Tables 1 and 2).

**Table 1:** Characteristics of type 2 diabetes and hypertension studies included in the review

Reference	Study design	Country, (province or region)	N	Sex	Age	Dietary Exposure	Dietary comparator	Health outcome
Masilela 2020 [46]	Cross-sectional	South Africa, (Mpumalanga)	157	24 males 133 females	Age categories: ≤ 49 years 50–59 years ≥ 60 years	Fruits and vegetables frequency of consumption (e.g., 1–3 times/week)	Fast food	Fasting blood samples were collected to measure: HbA1c levels
Galbete 2018 [48]	Cross-sectional	Ghana, (Ashanti)	2362 Rural: 942 Urban: 1420	Rural: 365 males 577 females Urban: 409 males 1011 females	Mean age: 46.2 ± 11.0 years Rural mean age:46.8 years Urban mean age: 45.3 years	“Roots, tubers and plantain” dietary pattern: refined cereals, fruits, nuts/seeds, roots/tubers/plantain, fermented maize products, legumes, palm oil and condiments.	“Mixed” dietary pattern “Rice, pasta, meat and fish” dietary pattern	Fasting blood samples were collected to measure: Glucose levels
Chikowore 2017 [49]	Cohort	South Africa, (North West)	2010	Rural: 347 males 659 females Urban: 399 males 605 females	Age range: 35 to 60 years	“Magnesium, phosphorus and plant protein” pattern “Starch, dietary fibre and B vitamin” pattern “Thiamine, zinc and plant protein” pattern “Thiamine, starch and folate” pattern	“Fat and animal protein” pattern “Retinol and vitamin B12” pattern “Beta carotene and vitamin C” pattern	Fasting blood samples were collected to measure: Glucose levels HbA1c levels
Kimani 2019 [44]	Cross-sectional	Kenya, (Nairobi)	229	102 males 127 females	Age categories: 18–25 years 26–35 years 36–45 years 46–55 years 56–65 years ≥ 66 years	Fruits and vegetables frequency of consumption (e.g., daily)	Fast food and meals high in animal fat	Blood pressure readings were taken. The Seventh Report of the Joint National Committee on Prevention, Detection, Evaluation, and Treatment of High Blood Pressure (JNC 7) classification was used.
Rush 2018 [42]	Cross-sectional	Zambia, (Western Province)	261 Urban:131 Rural: 130	92 males 169 females	Urban mean age: 39.40 ± 14.39 years Rural mean age: 54.13 ± 17.56 years	Fruit and vegetable frequency of consumption (e.g., average number of days/week)	–	Blood pressure readings were taken. The 2014 Clinical Practice Guidelines for the Management of Hypertension in the Community was used.
Katalambula 2017 [43]	Cross-sectional	Tanzania, (Arusha)	549	233 males 316 females	Mean age: 40.70 ± 12.07 years	‘Healthy’ dietary pattern: carbohydrate rich foods, vegetable, fruits, and spices. ‘Complex carbohydrate’ dietary pattern: sweets, roots, tubers, and legumes.	‘Western’ dietary pattern	Blood pressure readings were taken. The JNC 7 classification was used to define hypertension.

*N =* Sample size

**Table 2:** Characteristics of overweight/obesity, dyslipidaemia, metabolic syndrome, and acute stroke studies included in the review

Reference	Study design	Country, (province or region)	N	Sex	Age	Dietary Exposure	Dietary comparator	Health outcome
Motswagole 2020 [52]	Cross-sectional	Botswana, (nationwide)	1117	Only females	Mean age: 37.7 ± 16.3 years	Vegetable and fruit pattern: vegetables, fruits, fruit juices, and yellow/orange vegetables. Fish and nut pattern: fish, nuts, and cruciferous vegetables. Botswana traditional food pattern: insects (Phane), green leafy vegetables, legumes, and starches.	Fast food pattern Refined carbohydrate pattern Organ and red meat pattern	Overweight: BMI ≥25 kg/m^2^ Obesity: BMI ≥30 kg/m^2^
Darebo 2019 [53]	Cross-sectional	Ethiopia, (Southern Nations Nationalities Peoples)	524	230 males 294 females	Age range: 18 to 64 years	Fruits and vegetables frequency of consumption (e.g., 3–6 times/week)	Sweets, fast foods, meat, and egg	Overweight: BMI ≥25 kg/m^2^ Obesity: BMI ≥30 kg/m^2^
Gradidge 2018 [51]	Cross-sectional	South Africa, (Gauteng)	110	Only females	Mean age: 21.4 ± 3.29 years	Weekly food purchases of vegetables, salads, and fruits	Fish, meats, and fried foods	Overweight: BMI ≥25 kg/m^2^ Obesity: BMI ≥30 kg/m^2^
de Villiers 2018 [41]	Cross-sectional	South Africa, (Western Cape)	175	20 males 155 females	Median age: Men 36 years Women 39 years	Frequency of consumption of fruit and vegetables, brown bread, legumes, and high fibre cereals (e.g., times/day)	White bread and starches, High-fat foods, energy-dense snacks/items, and processed food	Overweight: BMI ≥25 kg/m^2^ Obesity: BMI ≥30 kg/m^2^
Holmes 2018 [47]	Cross-sectional	Nigeria, Tanzania, South Africa, and Uganda	738	294 males 444 females	Mean age: Men 38.6 ± 13.4 years Women 38.8 ± 13.0 years	Mixed dietary pattern: vegetables, fruits, cereals, nuts, beans, tea, diet soda, fruit juice, fresh fish, cold cuts, salad dressing, canned fish, and refined grains.	Processed dietary pattern	Overweight: BMI ≥25 kg/m^2^ Obesity: BMI ≥30 kg/m^2^
Otang-Mbeng 2017 [40]	Cross-sectional	South Africa, (Eastern Cape)	118	54 males 64 females	Age range: 21 to 70 years	Fruits and vegetables consumption (e.g., always)	Fast food	Overweight: BMI ≥25 kg/m^2^ Obesity: BMI ≥30 kg/m^2^
Njelekela 2002 [45]	Cross-sectional	Tanzania, (Dar es Salaam, Tanga and Arusha)	545	259 males 286 females	Age range: 46 to 58 years	Green vegetables and coconut milk frequency of consumption (e.g., days/week)	Whole milk, fish, and meat	Obesity: BMI ≥30 kg/m^2^ Dyslipidaemia: (TC-HDL-C)/HDL-C > 5
Mvitu Muaka 2010 [50]	Cross-sectional	DRC, (Kinshasa)	244	106 males 138 females	Mean age: 48 ± 16 years	Frequency of consumption of Cassava leaves, dried red beans, Solo and Bitekuteku (e.g., “regular” and portion/day)	–	MetS defined using: IDF criteria for Europids, NCEP-ATP III definition, and Modified IDF criteria specific for Central Africa.
O’Donnell 2016 [39]	Case-control	Mozambique, Nigeria, South Africa, Sudan, and Uganda	1949	Cases: 503 males 470 females Controls: Not reported	Mean age: 58.7 ± 15.2 years	Modified Alternative Healthy Eating Index	–	Acute stroke cases were identified using a computerized tomography or magnetic resonance imaging scans. The WHO criteria were used to define a stroke.

*N =* Sample size

### Plant-based dietary exposures identified in the African setting

Across the fifteen studies, the following plant-based dietary exposures were identified (see Additional file 4: Table S3): the consumption of plant foods, indicator food categories, adhering to a healthy dietary index and plant-based dietary or nutrient patterns.

### Consumption of plant foods

Eight of the included studies reported on the consumption of healthy plant foods. Fruits and vegetables were the most frequently reported plant foods [40, 42, 44, 46, 51, 53]. In addition to assessing the consumption of fruits and vegetables, we regarded the consumption of salads as a healthy plant food [51]. Other included studies assessed the usual consumption of vegetables with a high antioxidant content: leafy vegetables such as Cassava leaves (Manihot esculanta) and Bitetkuteku (*Amaranthus Hybridus*), and fruit vegetables and legumes namely Solo (*Solanum aethiopicum*) and dried red beans (*Phaseolus vulgaris*) [50], as well as green vegetables and coconut milk [45].

### Indicator food categories

One study reported on the assessment of six indicator food categories of which 3 met the criteria of our dietary exposure; the categories comprised of healthy plant foods such as fruits, vegetables, and legumes. These indicator food categories were identified using a food frequency questionnaire [41].

### Dietary index

A multi-country case-control study that included five African countries, applied the modified Alternative Healthy Eating Index (mAHEI) as a measure of dietary quality and investigated its relation to the incidence of stroke. The mAHEI includes the primary components of a PBD such as fruits, vegetables, wholes, nuts, and legumes [39].

### Plant-based dietary and nutrient patterns

Thirty-three percent of the studies reported on dietary patterns with higher factor loadings of plant versus animal foods. We classified these five studies as having plant-based dietary exposure patterns. Galbete et al., reported on the “Roots, tubers and plantain” dietary pattern, which was identified in their rural Ghanaian participants using principal component analysis (PCA) [48]. The PCA method was also applied by Holmes and colleagues who identified a “Mixed” dietary pattern, which differed slightly by sex. Three plant-based dietary patterns were identified in women from urban and rural areas in Botswana. Motswagole et al., labelled them as the: “Fruit and vegetable”, “Fish and nuts” and “Botswana traditional foods” dietary patterns [52]. Another study identified the “Healthy” and “Complex carbohydrate” dietary patterns that had high factor loadings for fruits, vegetables as well as legumes, roots, and tubers [43]. One study identified four plant driven nutrient patterns namely the “Magnesium, Phosphorus and Plant protein” and “Starch, Dietary Fibre and Vitamin B” driven patterns that was identified in rural Black South African women, and the “Thiamine, Starch and Folate” driven pattern in urban women. In addition to, the “Thiamine, Zinc and Plant Protein” pattern that was identified in their male counterparts [49].

### CVD risk outcomes and measures of association

All included studies reported on CVD/CVD risk profile as a health outcome: acute stroke (*n* = 1), dyslipidaemia (*n =* 1), MetS (*n =* 1), hypertension (*n* = 3), T2D (*n =* 3), and overweight/obesity (*n* = 7) and assessed its relation to plant-based dietary exposures. Most of the studies reported their effect estimate using ORs [42, 47, 48, 50, 53], and others reported the ARRs [43], relative risk (RR) [52] and population attributable risk (PAR) [39]. Katalambula et al., was the only study to present their data using ARRs [43]. Therefore, based on common statistical terminology and for the purpose of interpreting their results we assumed that it represents the absolute risk reduction (ARR) [55]. The association measures were presented with precision i.e., 95% confidence intervals (95% CIs), however, O’Donnell et al. was the only study that provided a narrower interval with 99% confidence [39]. Almost a third of the studies reported their data as prevalence estimates using count and percentage proportions [39, 40, 46, 47]. Three studies reported means with [51] or without standard deviations [44] and/or medians with the interquartile ranges [41]. Even fewer studies (*n* = 2) reported β regression coefficients with 95% CIs [45, 49]. A summary of the abovementioned reported effect estimates was recorded (see Additional file 4: Table S3).

### Association between plant-based dietary exposures and CVD risk profile in SSA

None of the included studies in this review stated that they assessed a PBD per se; however, they either assessed specific plant foods, food groups and/or dietary and nutrient patterns that were consistent with our dietary exposure of interest. Our results are therefore, presented according to the assessment of plant-based dietary exposures and their potential benefits to protect African populations against CVD. Nine studies had significant associations between the plant-based dietary exposures and CVD risk factors, this is outlined in Table S3 as part of the supplementary material (see Additional file 4). However, there was no significant association between hard CVD and the plant-based dietary exposure. In addition to this, no studies reported data on factors, which may alter the associations that were found between the plant-based dietary exposures and CVD risk profile across African populations. Below we have outlined the results and analyses performed by the studies that reported significant findings.

### Plant-based dietary exposures and type 2 diabetes (T2D)

The RODAM study investigated different dietary patterns among Ghanaian adults in relation with T2D risk. Only after conducting sensitivity analyses that excluded individuals with self-reported diabetes, did the authors find an inverse association (*p* = 0.016) with their plant-based dietary exposure. Rural Ghanaians had the highest adherence to the “roots, tubers and plantain” dietary pattern that was inversely associated with T2D, as presented for the highest tertile in adjusted model 3, the OR was 0.98 (95% CI:0.71–1.35) [48]. In South Africa, Chikowore et al. investigated which dietary factors are associated with predictive biomarkers of T2D. Two of the plant driven nutrient patterns that they identified: “Starch, dietary fibre and B vitamins” and the “Thiamine, zinc and plant protein” patterns, had significant inverse associations with the glycaemic markers in their rural participants. In men, the latter nutrient pattern inversely associated with low glycated haemoglobin (HbA1c) levels [β = − 0.288 (95% CI: − 0.543; − 0.033)], and fasting blood glucose levels [β = − 0.382 (95% CI: − 0.752; − 0.012)]. In rural women, who followed the “Starch, dietary fibre and B vitamins” driven nutrient pattern; the fasting glucose [β = − 0.236 (95% CI: − 0.458; − 0.014)] and HbA1c levels [β = − 0.175 (95% CI: − 0.303; − 0.047)] were significantly lower in the fully adjusted regression model [49].

### Plant-based dietary exposures and hypertension

Three studies found inverse associations between hypertension, and either the consumption of plant foods or a plant-based dietary pattern. Rush et al., reported significant rural vs. urban differences in the dietary intake of fruits amongst adults from Zambia (48.5% vs. 17.1%, *p* < 0.001). They found that eating more vegetables during the week was inversely associated with hypertension in rural Zambians, with an adjusted OR of 0.76 (95% CI:0.45–0.94) [42]. In Tanzania, Katalambula and colleagues conducted a study in residents of Arusha and showed that 42% of their participants consumed a ‘Healthy’ dietary pattern and were less likely to have hypertension. There was a significant negative association between the ‘Healthy’ dietary pattern and being hypertensive, the ARR was reported to be 0.82 (95% CI:0.68–0.99) [43]. The dietary practices of hypertensive patients from Kenya were assessed in relation to other risk factors. We observed that the authors of this study by Kimani et al., only reported crude association estimates (i.e., mean values) for their plant-based dietary exposures. In the latter study, the consumption of vegetables was significantly associated with a lower mean systolic blood pressure (SBP), for daily vs. frequently vs. rarely consuming vegetables and (*p*-values): 138.36 mmHg vs. 142.49 mmHg vs. 153.25 mmHg (0.032), lower diastolic blood pressure (DBP) 87.83 mmHg vs. 92.87 mmHg vs. 98.38 mmHg (*p* = 0.024), and lower body mass index (BMI) 28.55 kg/m^2^ vs. 29.85 kg/m^2^ vs. 34.36 kg/m^2^ (*p* = 0.006). The consumption of fruits daily, frequently and rarely was respectively associated with a lower mean BMI of 27.99 kg/m^2^, 29.68 kg/m^2^ and 29.79 kg/m^2^ (*p* = 0.011), and lower mean total cholesterol (TC) of 5.31 mmol/L, 5.71 mmol/L and 5.49 mmol/L (*p* = 0.033). The authors only conducted regression analysis for other risk factors i.e., BMI and alcohol intake, and did not report any adjusted association measures (i.e., ORs) for their dietary practices. As mentioned above, their dietary practices namely fruit and vegetable consumption appear to be inversely associated with hypertension based on the crude estimates [44].

### Plant-based dietary exposures and overweight/obesity

Three of the included studies reported significant negative associations between the consumption of plant foods and plant-based dietary patterns with overweight/obesity [40, 45, 51]. In the Eastern Cape province of South Africa, Otang-Mbeng and colleagues found that regularly eating vegetables was negatively associated with obesity (*p* < 0.05). However, the latter study did not perform regression analysis to determine whether adjusting for confounders such as older age and female sex, would affect the inverse association between vegetable consumption and obesity. Of note, this study had a quality score of 5 out of 14, which reflects poorly on its methodology [35]. In Tanzania, a study examined the association between obesity and the consumption of green vegetables and coconut milk among residents from three areas. Positive correlations were found between the consumption of coconut milk and BMI (*p* < 0.001), which was seen in both sexes. In addition to this, BMI status also correlated positively with the consumption of green vegetables (*p <* 0.001). Subsequently, when adjusting for confounders such as age in the multivariable regression analysis, it revealed that the positive association between the BMI status and coconut milk consumption only remained significant in males (*p* = 0.003) [45]. Motswagole et al., conducted a study in Botswana that identified six dietary patterns that were prevalent in females, and we regarded three of these patterns as being plant-based (Table 2). In their study there was a positive association between a high intake of the vegetable and fruit pattern and the risk of central obesity, RR of 1.43 (95% CI:1.18–1.72). Likewise, strong positive associations were seen in females with a higher adherence to the Botswana traditional food pattern and the risk of general (RR = 1.60, 95% CI:1.21–2.10) and central obesity (RR = 1.35, 95% CI:1.12–1.64). These positive associations were only maintained in individuals that were ranked as being highly adherent to the Botswana traditional food pattern (i.e., tertile 3); in spite of the further adjustments in their multivariate model that accounted for total energy intake [52].

### Plant-based dietary exposures and dyslipidaemia

Njelekela and colleagues also investigated the association between dyslipidaemia and dietary factors. They found a negative correlation between green vegetables consumption and TC in both males and females. However, these findings did not remained significant in their multivariable regression analyses that adjusted for age and BMI [45].

### Plant-based dietary exposures and metabolic syndrome (MetS)

One study investigated the association between antioxidant rich vegetables and a combination of CVD risk factors to determine the risk of MetS. This study was conducted among T2D patients in the DRC and its dietary focus was the regular consumption of vegetables rich in antioxidants. Only 13% of the study participants never consumed vegetables that are rich in antioxidants, and none of the T2D patients reported that they ate fruits. This study population mostly consumed Cassava leaves (39%), and dried red beans (26%) as their source of antioxidants, which were the only plant-based dietary exposures that was inversely associated with MetS after adjusting for confounders in their regression analysis. The consumption of Cassava leaves was found to have an independent protective effect on the risk of having MetS with an OR of 0.40 (95% CI:0.20–0.90). Similar inverse associations were reported in those consuming dried red beans, which was also protective against MetS with an OR of 0.40 (95% CI:0.20–0.80) [50].

### Quality assessment of the included studies

The quality of the 15 studies was assessed using the NHLBI tool for observational studies, the supplementary material shows the quality scores for each of the included studies (see Additional file 4: Table S2). The NHLBI quality assessment tool for cross-sectional and cohort studies consists of 14 questions, which were used to critically assess the methodology of 14 of the included studies. Only one study [39] was assessed using the NHLBI quality assessment tool for case-control studies that considered 12 criteria to rate the methodological quality. Fourteen studies were rated as having fair methodological quality, and one study [40] received a poor-quality rating. Two of the eligible studies [40, 41] did not meet at least 50% of the quality criteria as stipulated in the NHBLI tool for cross-sectional studies. Moreover, the majority of the included studies report cross-sectional findings that are prone to several biases and confounders [37]. Cross-sectional studies [56] are based on observations at a single timepoint, with insufficient power if the sample size is reasonably small [40, 51], which presents a poorer level of evidence [57]. Additionally, we cannot determine whether e.g., the identified plant-based dietary exposures are the cause or effect of the reduction in CVD risk factors, and vice versa. Causal relationships can only be deduced from longitudinal cohort observational and randomized intervention studies or case-control observational studies, where the effect/outcome is known [56].

We did not exclude any of the eligible studies based on quality, however, we have interpreted the results whilst considering its limitations. We identified minor instances of implausible reporting of study findings. In terms of the study characteristics specifically the demographics; two of the studies [44, 46] presented the age of their participants in a manner that was not easy to interpret. Kimani et al., reported age as a categorial variable by comparing three age groups, however, reporting the mean age would have given the readers an indication as to whether or not the age variable was normally distributed [44]. Masilela and others followed the same approach by only reporting the age of their participants categorically (Table 1). Although these are minor reporting biases, age is a well-known confounder when assessing associations, and should therefore be reported as clearly as possible. Masilela et al., appropriately adjusted for age during their logistic regression analysis [46]. However, Kimani and colleagues did not state whether they adjusted for age in their regression models and did not include all the relevant predictors. In the paper by Kimani et al., they did not state why they did not perform logistic regression analysis for their plant-based dietary exposures i.e., daily consumption of fruits and vegetables, which was statistically significant in their analysis of variance (ANOVA) tables [44].

## Discussion

In this systematic review, we assessed the association of plant-based dietary exposures and CVD risk in SSA. This study was conducted against the backdrop of the global [1] and regional [3] CVD burden in conjunction with the current phase (stage five) of the nutrition transition [9]; which encourages the consumption of healthy “plant-based” foods and discourages the intake of highly processed foods. We identified fifteen studies that were conducted in 12 SSA countries and collated data from: East [43–45, 53], South [40–42, 46, 49, 51, 52], West [48], Central [50] Africa and across multiple regions [39, 47]. These studies have primarily focused on the frequency of consumption of plant foods as a plant-based dietary exposure and mainly assessed its association with overweight/obesity. Some studies utilized data driven analysis to assess the adherence to plant-based dietary and nutrient patterns. One study [39] investigated the dietary intake of African populations using a predefined dietary index, which consists of plant foods that are associated with health outcomes.

This review presents evidence that some plant-based dietary exposures are significantly associated with CVD risk factors in African populations. We observed that 9 of the 15 included studies reported inverse [40, 42–44, 48–50] and positive [45, 52] associations between a plant-based dietary exposure and CVD risk as a health outcome. Dietary practices that predominantly consist of plant foods [28] have previously been described among African populations in the early 1990s [58]. More than half (53%) of the included studies in this review investigated the frequency of consumption of plant foods, therefore, mainly reporting on the components of a PBD i.e., fruits and vegetables [40, 42, 44–46, 50, 51, 53]. Five of the latter studies reported statistically significant associations with the following CVD risk factors: hypertension [42, 44], metabolic syndrome [50], obesity and dyslipidaemia [45]. However, unhealthy dietary habits continue to prevail in SSA, whereby 72% of adults, do not meet the daily recommended fruit and vegetable intake [59]. A possible limitation of the latter studies is that they solely assessed the associations of single plant foods that were usually consumed within a specific study population e.g., female university students [51] or known diabetics [50] within a particular province/region of a country. Nonetheless, fruits and vegetables have been extensively researched for their health benefits conferred by nutrients such as vitamins, minerals, polyphenols, dietary fibre, and antioxidants, which protect against CVD and other chronic diseases [13, 29].

Dietary pattern studies consider the intake of both plant and animal foods. Our focus was on the health outcomes that were associated with plant-based dietary exposures. Five studies assessed plant-based dietary and nutrient patterns [43, 47–49, 52], and 4 of these studies were significantly inversely associated with hypertension [43], T2D [48, 49] and positively associated with overweight/obesity [52]. For instance, the South African arm of the PURE cohort study [49] found that the consumption of nutrient patterns with higher factor loadings for proteins, carbohydrates and dietary fibre sourced from plants provided protection against T2D in rural men and women. These plant-based dietary and nutrient pattern studies differ from the above-mentioned studies that assessed single plant foods [40, 42, 44–46, 50, 51, 53], in that they applied data driven analysis using PCA to identify patterns and investigate the associations between the overall diet of their study population and CVD risk.

The quality of a diet can be assessed by calculating a dietary index/score to determine the adherence to healthy diets i.e., the Mediterranean or dietary approaches to stop hypertension (DASH) diet scores, as well as the Healthy Eating Index (HEI) [60]. Only one study [39] utilized a dietary index namely the mAHEI, which was constructed using food components derived from plant and animal sources that are predictive of chronic disease. Obtaining a high score when using the mAHEI suggests a reduced risk of developing chronic diseases e.g., CVD risk factors or CVD. Its scoring method scores each food component a score from 0 to 10 [60], which notably resembles the plant-based dietary indices (PDIs) that applies a reverse grading system [22]. The mAHEI accounts for the total dietary intake and scores the food components accordingly which better reflects the overall diet compared to single foods [61, 62]. There were no significant associations between the mAHEI that was utilized to score the diet quality of five African populations (OR, 0.78) at probable risk of acute stroke [39]. However, all the other regions included in the international study by O’Donnell and others namely in the Americas, Australia, Europe, and Asia (expect for South Asia) had a significant inverse association between diet and stroke. Of note, in comparison to the other regions Africa had much stronger associations with the other modifiable CVD risk factors of stroke such as hypertension (OR, 4.01), alcohol consumption (OR, 5.91) and physical inactivity (OR, 0.95). Apart from the latter multi-country case-control study [39], we did not find other African studies that applied the predefined dietary analysis approach to assess plant-based dietary exposures with CVDs.

Six (40%) of the included studies had no significant associations between plant-based dietary exposures and CVD risk profile, which comprises 3653 participants that contributed to the total sample size of this review. The latter could be due to a number of reasons: small sample size [41, 46, 51], confounding effects e.g., higher intakes of alcohol and/or dietary comparators namely processed [47] and fast foods [53], which may counteract the protective effects of plant-based dietary exposures. The adoption of Westernized diets that largely promote the consumption of processed foods in Africa [8] and other low-to-middle income countries [63], may explain why O’Donnell [39] reported no significant associations between acute stroke and the mAHEI or the consumption of plant foods namely fruit and vegetables for the African region. Despite, evidence that the nutrition transition is occurring in SSA, challenges such as urbanization and modernization of cultures still adversely influence dietary habits.

Amidst the nutrition transition in SSA [59], there are also existing health disparities between and within the 12 SSA countries that were included in this review, i.e., poverty that contributes to their disease susceptibility. Based on the World Bank data country classifications [54], most of the 12 SSA countries are classified as being low-to-middle income [42–45, 47, 48, 50]. Considering the latter, we recommend that the social determinants of health (SDOH) [64], in particular the economic stability of these study populations amongst other SDOH such as behavioural and environmental risk factors should not be ignored when interpreting the results of this review. It is also worth noting that according to the Food and Agriculture Organization of the United Nation’s [65], only 3 of the 12 SSA countries namely Kenya, Nigeria and South Africa have food-based dietary guidelines (FBDGs). Therefore, in the absence of country specific FBDGs, thought should also be given to impact that behavioural risk factors i.e., unhealthy dietary habits may have on the growing incidence of NCDs such as CVD in African populations [2, 59].

A recent review showed that there is a lack of nutritional epidemiological and clinical studies such as randomized controlled trials (RCTs) in low-to-middle income countries [24]. While we acknowledge that there are challenges faced when conducting nutrition trials e.g. no control group or a lack of participant adherence [66], it should be taken into consideration that the evidence-based literature on the effects of PBDs and CVD risk, is presently based on RCTs that are predominantly from high income countries [24]. McMacken & Shah [67] reviewed the prevention of T2D by means of consuming plant-based eating patterns. In the latter review they utilized data from observational cohort studies, which were conducted in high income countries in North America [27, 68–70] and East Asia [71] to examine the health benefits of a PBD in relation to T2D. The lack of evidence on this research topic from SSA amongst other low-to-middle income countries, remain a concern and greater efforts are needed to address this gap in the literature.

### Strengths and limitations

To our knowledge, this is the first systematic review to collate data on assessing the association between plant-based dietary exposures and CVD risk profile in African populations. A major finding of this systematic review was that none of the studies that were included stated that they assessed a PBD per se. Therefore, the current evidence which we collated is not suitable for assessing the associations of an overall PBD in African populations with CVD risk profile. As a strength, this systematic review describes four plant-based dietary exposures identified across twelve SSA countries and presents evidence on its associations with CVD risk factors. Two of these plant-based dietary exposures namely: consumption of plant foods and the adherence to a plant-based dietary or nutrient pattern had significant associations with CVD risk profile in the African population. However, the accuracy and precision of these dietary exposures, which were mainly single plant foods and/or PBD components can be improved upon. One of the study limitations was that very few studies; 15 of the 458 retrieved articles met our inclusion criteria. Due to the substantial amount of heterogeneity between the included studies regarding their: reported dietary exposures, effect estimates and/or variances, we were unable to perform meta-analyses. We also acknowledge that we were unable to deduce any causal relationships from the cross-sectional studies that were included in this systematic review. Future studies from SSA should focus on assessing an overall PBD and aim to standardize the assessment of PBDs on the African continent. Subsequently, this could inform clinicians and policymakers [72] by providing them with a better understanding of what a PBD entails. In addition to, promoting healthy eating habits i.e., PBD adherence to prevent CVD risk factors and alleviate the NCD burden in under resourced African healthcare settings.

## Conclusions

This review has showed that the knowledge gap on the association between PBDs and CVD risk profile in Africa is still prominent. Although there is substantiating evidence of plant-based dietary exposures being associated with clinical measurements of CVD risk reduction i.e., a lower likelihood of African populations being obese or hypertensive and lower biomarker levels i.e., HbA1c in diabetics. Further investigations are needed to explore the inverse associations between plant-based dietary exposures and CVD risk profile. However, these associations imply that African populations in urban and rural areas should respectively, revert to and maintain the consumption of traditional diets with an abundance of minimally processed plant foods to potentially prevent NCDs e.g., T2D and CVD.

## Supplementary Information

**Additional file 1.** PRISMA 2020 Checklist

**Additional file 2.** Search strategies

**Additional file 3.** Excel Workbook with exclusion reasons

**Additional file 4:** **Table S2.** Quality assessment of observational studies using the NHLBI tools. **Table S3.** Plant-based dietary exposures identified across studies conducted in SSA between 2002 and 2020

## Data Availability

The data supporting the conclusions of this study are included in this published article and its supplementary information files.

## Abbreviations

AJOL: African Journals Online
ANOVA: Analysis of variance
ARR: Absolute risk reduction
BMI: Body mass index
CPCI: Conference Proceedings Citation Index
CVD: Cardiovascular disease
DASH: Dietary Approaches to Stop Hypertension
DM: Diabetes mellitus
DRC: Democratic Republic of the Congo
HbA1c: Glycated haemoglobin
HDL-C: High-density lipoprotein cholesterol
HEI: Healthy Eating Index
JNC 7: The Seventh Report of the Joint National Committee on Prevention, Detection, Evaluation, and Treatment of High Blood Pressure
LDL-C: Low-density lipoprotein cholesterol
mAHEI: modified Alternative Healthy Eating Index
MetS: Metabolic syndrome
NCD: Non-communicable disease
NHLBI: National Heart, Lung, and Blood Institute
OR: Odds ratio
PAR: Population attributable risk
PBD: Plant-based diet
PCA: Principal component analysis
PDIs: Plant-based dietary indices
PECO: Population, Exposure, Comparator for the exposure and desired Outcome
PRISMA: Preferred Reporting Items for Systematic Reviews and Meta-Analyses
PROSPERO: The International Prospective Register of Systematic Reviews
RCTs: Randomized controlled trials
RR: Relative risk
SDOH: Social determinants of health
SSA: sub-Saharan Africa
T2D: Type 2 diabetes
TC: Total cholesterol
TIABs: Titles and abstracts
WHO: World Health Organization

## References

[1] World Health Organization (WHO). The top 10 causes of death. Available at https://www.who.int/newsroom/fact-sheets/detail/the-top-10-causes-of-death. Accessed on 06 Dec 2021.

[2] Yuyun MF, Sliwa K, Kengne AP, Mocumbi AO, Bukhman G (2020). Cardiovascular diseases in sub-Saharan Africa compared to high-income countries: an epidemiological perspective. Glob Heart.

[3] World Health Organization (WHO). Atlas of African Health Statistics: The Sustainable development goals and Universal health coverage in the WHO African Region. World Health Organization. Regional Office for Africa. 2019. p. 1–131. Accessed from www.aho.afro.who.int.

[4] International Diabetes Federation (IDF) Diabetes Atlas 9th ed.; 2019. p. 1–176. Accessed from https://diabetesatlas.org/atlas/ninth-edition/.

[5] Popkin BM (2004). The nutrition transition: an overview of world patterns of change. Nutr Rev.

[6] Popkin BM (1998). The nutrition transition and its health implications in lower-income countries. Public Health Nutr.

[7] Encyclopedia.com. “Famine.” Encyclopedia of Genocide and Crimes Against Humanity. Available at https://www.encyclopedia.com/international/encyclopedias-almanacs-transcripts-and-maps/famine. Accessed 06 Dec 2021.

[8] Reardon T, Tschirley D, Liverpool-Tasie LSO, Awokuse T, Fanzo J, Minten B, Vos R, Dolislager M, Sauer C, Dhar R (2021). The processed food revolution in African food systems and the double burden of malnutrition. Glob Food Sec.

[9] Abrahams Z, McHiza Z, Steyn NP (2011). Diet and mortality rates in sub-Saharan Africa: stages in the nutrition transition. BMC Public Health.

[10] Vorster HH (2002). The emergence of cardiovascular disease during urbanisation of Africans. Public Health Nutr.

[11] Magkos F, Tetens I, Bugel SG, Felby C, Schacht SR, Hill JO, Ravussin E, Astrup A: A perspective on the transition to plant-based diets: a diet change may attenuate climate change, but can it also attenuate obesity and chronic disease risk? Adv Nutr 2020, 11(1):1–9. 10.1093/advances/nmz090 PMC7442415 31504086

[12] Willett W, Rockström J, Loken B, Springmann M, Lang T, Vermeulen S, Garnett T, Tilman D, DeClerck F, Wood A (2019). Food in the Anthropocene: the EAT–lancet commission on healthy diets from sustainable food systems. Lancet.

[13] Hu FB (2003). Plant-based foods and prevention of cardiovascular disease: an overview. Am J Clin Nutr.

[14] Massera D, Zaman T, Farren GE, Ostfeld RJ. A whole-food plant-based diet reversed angina without medications or procedures. Case Rep Cardiol. 2015;2015:1–3. 10.1155/2015/978906. PMC4338379 25755896

[15] Massera D, Graf L, Barba S, Ostfeld R (2016). Angina rapidly improved with a plant-based diet and returned after resuming a Western diet. J Geriatr Cardiol.

[16] Najjar RS, Moore CE, Montgomery BD (2018). A defined, plant-based diet utilized in an outpatient cardiovascular clinic effectively treats hypercholesterolemia and hypertension and reduces medications. Clin Cardiol.

[17] Najjar RS, Moore CE, Montgomery BD (2018). Consumption of a defined, plant-based diet reduces lipoprotein(a), inflammation, and other atherogenic lipoproteins and particles within 4 weeks. Clin Cardiol.

[18] Najjar RS, Montgomery BD (2019). A defined, plant-based diet as a potential therapeutic approach in the treatment of heart failure: a clinical case series. Complement Ther Med.

[19] Ostfeld RJ (2017). Definition of a plant-based diet and overview of this special issue. J Geriatr Cardiol.

[20] Williams KA, Patel H, Williams KA (2017). Healthy plant-based diet: what does it really mean?. J Am Coll Cardiol.

[21] Tuso P, Stoll SR, Li WW (2015). A plant-based diet, atherogenesis, and coronary artery disease prevention. Perm J.

[22] Satija A, Bhupathiraju SN, Spiegelman D, Chiuve SE, Manson JE, Willett W, Rexrode KM, Rimm EB, Hu FB (2017). Healthful and unhealthful plant-based diets and the risk of coronary heart disease in U.S. adults. J Am Coll Cardiol.

[23] Kim H, Rebholz CM, Garcia-Larsen V, Steffen LM, Coresh J, Caulfield LE (2020). Operational differences in plant-based diet indices affect the ability to detect associations with incident hypertension in middle-aged US adults. J Nutr.

[24] Toumpanakis A, Turnbull T, Alba-Barba I (2018). Effectiveness of plant-based diets in promoting well-being in the management of type 2 diabetes: a systematic review. BMJ Open Diabetes Res Care.

[25] Hemler EC, Hu FB (2019). Plant-based diets for cardiovascular disease prevention: all plant foods are not created equal. Curr Atheroscler Rep.

[26] Molina-Montes E, Salamanca-Fernández E, Garcia-Villanova B, Sánchez MJ (2020). The impact of plant-based dietary patterns on cancer-related outcomes: a rapid review and meta-analysis. Nutrients.

[27] Satija A, Bhupathiraju SN, Rimm EB, Spiegelman D, Chiuve SE, Borgi L, Willett WC, Manson JE, Sun Q, Hu FB (2016). Plant-based dietary patterns and incidence of type 2 diabetes in US men and women: results from three prospective cohort studies. PLoS Med.

[28] Valdes M, Conklin A, Veenstra G, Black JL (2021). Plant-based dietary practices in Canada: examining definitions, prevalence and correlates of animal source food exclusions using nationally representative data from the 2015 Canadian Community Health Survey–Nutrition. Public Health Nutr.

[29] Salas-Salvadó J, Becerra-Tomás N, Papandreou C, Bulló M (2019). Dietary patterns emphasizing the consumption of plant foods in the management of type 2 diabetes: a narrative review. Adv Nutr.

[30] Page MJ, McKenzie JE, Bossuyt PM, Boutron I, Hoffmann TC, Mulrow CD, Shamseer L, Tetzlaff JM, Akl EA, Brennan SE (2021). The PRISMA 2020 statement: an updated guideline for reporting systematic reviews. BMJ.

[31] Morgan RL, Whaley P, Thayer KA, Schunemann HJ (2018). Identifying the PECO: a framework for formulating good questions to explore the association of environmental and other exposures with health outcomes. Environ Int.

[32] Vega Mejía N, Ponce Reyes R, Martinez Y, Carrasco O, Cerritos R (2018). Implications of the Western diet for agricultural production, health and climate change. Front Sustain Food Syst.

[33] Unger T, Borghi C, Charchar F, Khan NA, Poulter NR, Prabhakaran D, Ramirez A, Schlaich M, Stergiou GS, Tomaszewski M (2020). 2020 International Society of Hypertension Global Hypertension Practice Guidelines. Hypertension.

[34] World Health Organization/International Diabetes Federation (WHO/IDF). Definition and diagnosis of diabetes and intermediate hyperglycaemia: report of a WHO/IDF consultation. 2006. p. 1–50. Accessed from https://apps.who.int/iris/handle/10665/43588.

[35] Lopes T, Zemlin AE, Erasmus RT, Faber M, Kengne AP (2020). Assessment of the association of plant-based diets with cardiovascular disease risk profile in Africa: a systematic review and meta-analysis protocol. BMJ Open.

[36] Kufe NC, Masemola M, Chikowore T, Kengne AP, Olsson T, Goedecke JH, Micklesfield LK (2019). Protocol for systematic review and meta-analysis of sex hormones and diabetes risk in ageing men and women of African ancestry. BMJ Open.

[37] National Heart, Lung, and Blood Institiute (NHLBI). Assessing Cardiovascular Risk: Systematic Evidence Review from the Risk Assessment Work Group, evidence report. 2013. p. 1–139. Accessed from https://www.nhlbi.nih.gov/sites/default/files/media/docs/risk-assessment.pdf.

[38] Mendez-Bustos P, Calati R, Rubio-Ramirez F, Olie E, Courtet P, Lopez-Castroman J (2019). Effectiveness of psychotherapy on suicidal risk: a systematic review of observational studies. Front Psychol.

[39] O'Donnell MJ, Chin SL, Rangarajan S, Xavier D, Liu L, Zhang H, Rao-Melacini P, Zhang X, Pais P, Agapay S (2016). Global and regional effects of potentially modifiable risk factors associated with acute stroke in 32 countries (INTERSTROKE): a case-control study. Lancet.

[40] Otang-Mbeng W, Otunola GA, Afolayan AJ (2017). Lifestyle factors and co-morbidities associated with obesity and overweight in Nkonkobe municipality of the eastern cape, South Africa. J Health Popul Nutr.

[41] de Villiers A, Senekal M, Nel J, Draper CE, Lambert E, Steyn NP (2018). The HealthKick study: modifiable lifestyle factors in primary caregivers of primary school learners from two school districts in the Western Cape Province, South Africa. Ethn Dis.

[42] Rush KL, Goma FM, Barker JA, Ollivier RA, Ferrier MS, Singini D (2018). Hypertension prevalence and risk factors in rural and urban Zambian adults in western province: a cross-sectional study. Pan Afr Med J.

[43] Katalambula LK, Meyer DN, Ngoma T, Buza J, Mpolya E, Mtumwa AH, Petrucka P (2017). Dietary pattern and other lifestyle factors as potential contributors to hypertension prevalence in Arusha City, Tanzania: a population-based descriptive study. BMC Public Health.

[44] Kimani S, Mirie W, Chege M, Okube OT, Muniu S (2019). Association of lifestyle modification and pharmacological adherence on blood pressure control among patients with hypertension at Kenyatta National Hospital, Kenya: a cross-sectional study. BMJ Open.

[45] Njelekela M, Kuga S, Nara Y, Ntogwisangu J, Masesa Z, Mashalla Y, Ikeda K, Mtabaji J, Yamori Y, Tsuda K (2002). Prevalence of obesity and dyslipidemia in middle-aged men and women in Tanzania, Africa: relationship with resting energy expenditure and dietary factors. J Nutr Sci Vitaminol.

[46] Masilela C, Pearce B, Ongole JJ, Adeniyi OV, Benjeddou M (2020). Factors associated with glycemic control among south African adult residents of Mkhondo municipality living with diabetes mellitus. Medicine.

[47] Holmes MD, Dalal S, Sewram V, Diamond MB, Adebamowo SN, Ajayi IO, Adebamowo C, Chiwanga FS, Njelekela M, Laurence C (2018). Consumption of processed food dietary patterns in four African populations. Public Health Nutr.

[48] Galbete C, Nicolaou M, Meeks K, Klipstein-Grobusch K, De-Graft Aikins A, Addo J, Amoah SK, Smeeth L, Owusu-Dabo E, Spranger J (2018). Dietary patterns and type 2 diabetes among Ghanaian migrants in Europe and their compatriots in Ghana: the RODAM study. Nutr Diabetes.

[49] Chikowore T, Pisa PT, van Zyl T, Feskens EJ, Wentzel-Viljoen E, Conradie KR (2017). Nutrient patterns associated with fasting glucose and glycated haemoglobin levels in a Black south African population. Nutrients.

[50] Mvitu Muaka M, Longo-Mbenza B, Tulomba Mona D, Nge Okwe A (2010). Reduced risk of metabolic syndrome due to regular intake of vegetables rich in antioxidants among African type 2 diabetics. Diabetes Metab Syndr Clin Res Rev.

[51] Gradidge PJ-L, Cohen E (2018). Body mass index and associated lifestyle and eating behaviours of female students at a south African university. South Afr J Clin Nutr.

[52] Motswagole B, Jackson, J, Kobue-Lekalake R, Maruapula S, Mongwaketse T, Kwape L, Thomas T, Swaminathan S, Kurpad AV, Jackson M. The association of general and central obesity with dietary patterns and socioeconomic status in adult women in Botswana. J Obes. 2020;2020:1–10. 10.1155/2020/4959272. 10.1155/2020/4959272 PMC7491446 32963826

[53] Darebo T, Mesfin A, Gebremedhin S (2019). Prevalence and factors associated with overweight and obesity among adults in Hawassa city, southern Ethiopia: a community based cross-sectional study. BMC Obes.

[54] The World Bank. World Bank Country and Lending Groups | Country classification. Available at https://datahelpdesk.worldbank.org/knowledgebase/articles/906519-world-bank-country-and-lending-groups. Accessed 10 Dec 2021.

[55] Schechtman E (2002). Odds ratio, relative risk, absolute risk reduction, and the number needed to treat—which of these should we use?. Value Health.

[56] Mann CJ (2003). Observational research methods. Research design II: cohort, cross sectional, and case-control studies. Emerg Med J.

[57] Murad MH, Asi N, Alsawas M, Alahdab F (2016). New evidence pyramid. Evid Based Med.

[58] Famodu AA, Osilesi O, Makinde YO, Osonuga OA (1998). Blood pressure and blood lipid levels among vegetarian, semi-vegetarian, and non-vegetarian native Africans. Clin Biochem.

[59] Nnyepi MS, Gwisai N, Lekgoa M, Seru T (2015). Evidence of nutrition transition in southern Africa. Proc Nutr Soc.

[60] Chiuve SE, Fung TT, Rimm EB, Hu FB, McCullough ML, Wang M, Stampfer MJ, Willett WC (2012). Alternative dietary indices both strongly predict risk of chronic disease. J Nutr.

[61] Burggraf C, Teuber R, Brosig S, Meier T (2018). Review of a priori dietary quality indices in relation to their construction criteria. Nutr Rev.

[62] Trijsburg L, Talsma EF, de Vries JHM, Kennedy G, Kuijsten A, Brouwer ID (2019). Diet quality indices for research in low- and middle-income countries: a systematic review. Nutr Rev.

[63] Stuckler D, McKee M, Ebrahim S, Basu S (2012). Manufacturing epidemics: the role of global producers in increased consumption of unhealthy commodities including processed foods, alcohol, and tobacco. PLoS Med.

[64] Eshetu EB, Woldesenbet SA (2011). Are there particular social determinants of health for the world's poorest countries?. Afr Health Sci.

[65] Food and Agriculture Organization of the United Nations (FAO). Food-based dietary guidelines in Africa. Available at https://www.fao.org/nutrition/education/food-dietary-guidelines/regions/africa/en/. Accessed on 09 Dec 2021.

[66] Laville M, Segrestin B, Alligier M, Ruano-Rodríguez C, Serra-Majem L, Hiesmayr M, Schols A, La Vecchia C, Boirie Y, Rath A (2017). Evidence-based practice within nutrition: what are the barriers for improving the evidence and how can they be dealt with?. Trials.

[67] McMacken M, Shah S (2017). A plant-based diet for the prevention and treatment of type 2 diabetes. J Geriatr Cardiol.

[68] Tonstad S, Butler T, Yan R, Fraser GE (2009). Type of vegetarian diet, body weight, and prevalence of type 2 diabetes. Diabetes Care.

[69] Tonstad S, Stewart K, Oda K, Batech M, Herring RP, Fraser GE (2013). Vegetarian diets and incidence of diabetes in the Adventist health Study-2. Nutr Metab Cardiovasc Dis.

[70] Vang A, Singh PN, Lee JW, Haddad EH, Brinegar CH (2008). Meats, processed meats, obesity, weight gain and occurrence of diabetes among adults: findings from Adventist health studies. Ann Nutr Metab.

[71] Chiu TH, Huang HY, Chiu YF, Pan WH, Kao HY, Chiu JP, Lin MN, Lin CL (2014). Taiwanese vegetarians and omnivores: dietary composition, prevalence of diabetes and IFG. PLoS One.

[72] Janse Van Rensburg LM. Wiles NL: the opinion of KwaZulu-Natal dietitians regarding the use of a whole-foods plant-based diet in the management of non-communicable diseases. South Afr J Clin Nutr. 2019:1–5.

