# Supplementary material for: Assessment of the association between plant-based dietary exposures and cardiovascular disease risk profile in sub-Saharan Africa: a systematic review
Source: BMC Public Health. 2022 Feb 19;22:361. doi: 10.1186/s12889-022-12724-w (PMC8858494; doi:10.1186/s12889-022-12724-w)
Supplement: Supplementary file 2 — Additional file 2. Search strategies [file 12889_2022_12724_MOESM2_ESM.docx]

**Additional file 2. Search strategies**

| **PubMed-MEDLINE** |
| --- |
| **#1 - POPULATION** |
| Africa* OR Algeria OR Angola OR Benin OR Botswana OR Burkina Faso OR Burundi OR Cameroon OR Cameroun OR Cape Verde OR Central African Republic OR République Centre Afrique OR RCA OR CAR OR Chad OR chad OR Comoros Islands OR Comoros OR Congo Or Democratic Republic of Congo OR DRC OR République Démocratique du Congo OR RDC OR Djibouti OR Egypt OR Equatorial Guinea OR Eritrea OR Ethiopia OR Gabon OR Gambia OR Ghana OR Guinea OR Guinea Bissau OR Ivory Coast OR Cote d’Ivoire OR Kenya OR Lesotho OR Liberia OR Libya OR Madagascar OR Malawi OR Mali OR Mauritania OR Mauritius OR Mayotte OR Morocco OR Mozambique OR Namibia OR Niger OR Nigeria OR Principe OR Sao Tome OR Sao Tome & Principe OR Rwanda OR Senegal OR Seychelles OR Sierra Leone OR Somalia OR Somali Land OR South Africa* OR South Sudan OR Sudan OR Swaziland OR Tanzania OR Togo OR Tunisia OR Uganda OR Western Sahara* OR Zambia OR Zimbabwe OR Central Africa* OR West Africa* OR Western Africa* OR East Africa* OR Eastern Africa* OR North Africa* OR Northern Africa* OR Southern Africa* OR sub Saharan Africa* OR sub-Saharan Africa* OR Africa South of Sahara* OR African descent OR African ancestry OR Africans |
| **#2 -** **EXPOSURE** |
| Healthy dietary patterns OR Plant-based diet OR Healthy diet OR Traditional diet OR Vegetarian diet  OR Vegan diet OR Mediterranean diet OR Dietary approaches to stop hypertension OR DASH diet OR  nutrition OR diet, vegetarian [MeSH Terms] OR diet, vegan [MeSH Terms] OR diets, vegetarian [MeSH  Terms] OR dietary habits [MeSH Terms] OR behaviors, eating [MeSH Terms] |
| **#3 - COMPARATOR** |
| Unhealthy plant dietary patterns OR Westernised diet OR Animal-based OR Fast foods OR Processed  foods |
| **#4 - HEALTH OUTCOMES** |
| Cardiovascular disease OR Metabolic syndrome OR Hypertension OR Diabetes mellitus OR  Insulin resistance OR Hyperglycaemia OR Dysglycaemia OR Prediabetes OR Dyslipidaemia OR  Hypercholesterolaemia OR Hypertriglyceridaemia OR Obesity OR Overweight |
| **#5** |
| #1 AND #2 AND #3 AND #4 |
| **FILTERS AND/OR LIMITS APPLIED** |
| Humans, English, French, Adults:19+ years |
| **Scopus** |
| **Query 1 - POPULATION** |
| africa* OR algeria OR angola OR benin OR botswana OR burkina AND faso OR burundi OR cameroon OR cameroun OR cape AND verde OR central AND african AND republic OR république AND centre AND afrique OR rca OR car OR chad OR chad OR comoros AND islands OR comoros OR congo OR democratic AND republic AND of AND congo OR drc OR république AND démocratique AND du AND congo OR rdc OR djibouti OR egypt OR equatorial AND guinea OR eritrea OR ethiopia OR gabon OR gambia OR ghana OR guinea OR guinea AND bissau OR ivory AND coast OR cote AND d'ivoire OR kenya OR lesotho OR liberia OR Libya OR madagascar OR malawi OR mali OR mauritania OR mauritius OR mayotte OR morocco OR mozambique OR namibia OR niger OR nigeria OR principe OR sao AND tome OR sao AND tome AND & AND principe OR rwanda OR senegal OR seychelles OR sierra AND leone OR somalia OR somali AND land OR south AND africa* OR south AND sudan OR sudan OR swaziland OR tanzania OR togo OR tunisia OR uganda OR western AND sahara* OR zambia OR zimbabwe OR central AND africa* OR west AND africa* OR western AND africa* OR east AND africa* OR eastern AND africa* OR north AND africa* OR northern AND africa* OR southern AND africa* OR sub AND saharan AND africa* OR sub-saharan AND africa* OR africa AND south AND of AND sahara* OR african AND descent OR african AND ancestry OR africans |
| **Query 2 -** **EXPOSURE** |
| traditional AND diet OR diet OR healthy AND dietary AND patterns OR healthy AND diet OR vegetarian AND diet OR vegetarian OR vegetarianism OR vegan AND diet OR vegan OR plant AND based AND diet OR plant AND foods OR mediterranean AND diet OR dietary AND approaches AND to AND stop AND hypertension OR dash AND diet OR dietary AND patterns OR eating AND behaviour OR nutrition |
| **Query 3 - COMPARATOR** |
| unhealthy AND dietary AND patterns OR westernized AND diet OR western AND diet OR animal AND based AND diet OR fast AND foods |
| **Query 4 - HEALTH OUTCOMES** |
| cardiovascular AND disease OR metabolic AND syndrome OR hypertension OR diabetes AND mellitus OR insulin AND resistance OR hyperglycemia OR dysglycaemia OR prediabetes OR dyslipidaemia OR hypercholesterolemia OR hypertriglyceridemia OR obesity OR overweight |
| **Query 5** |
| #1 AND #2 AND #3 AND #4 |
| **Query 6** |
| Association AND of AND plant-based AND diet AND cardiovascular AND disease AND risk AND in AND Africa |
| **FILTERS AND/OR LIMITS APPLIED** |
| (Publication year, 1990-2021); (Document type, article) |
| **EBSCOhost** |
| **S1 - POPULATION** |
| Africa* OR Algeria OR Angola OR Benin OR Botswana OR Burkina Faso OR Burundi OR Cameroon OR Cameroun OR Cape Verde OR Central African Republic OR République Centre Afrique OR RCA OR CAR OR Chad OR chad OR Comoros Islands OR Comoros OR Congo Or Democratic Republic of Congo OR DRC OR République Démocratique du Congo OR RDC OR Djibouti OR Egypt OR Equatorial Guinea OR Eritrea OR Ethiopia OR Gabon OR Gambia OR Ghana OR Guinea OR Guinea Bissau OR Ivory Coast OR Cote d’Ivoire OR Kenya OR Lesotho OR Liberia OR Libya OR Madagascar OR Malawi OR Mali OR Mauritania OR Mauritius OR Mayotte OR Morocco OR Mozambique OR Namibia OR Niger OR Nigeria OR Principe OR Sao Tome OR Sao Tome & Principe OR Rwanda OR Senegal OR Seychelles OR Sierra Leone OR Somalia OR Somali Land OR South Africa* OR South Sudan OR Sudan OR Swaziland OR Tanzania OR Togo OR Tunisia OR Uganda OR Western Sahara* OR Zambia OR Zimbabwe OR Central Africa* OR West Africa* OR Western Africa* OR East Africa* OR Eastern Africa* OR North Africa* OR Northern Africa* OR Southern Africa* OR sub Saharan Africa* OR sub-Saharan Africa* OR Africa South of Sahara* OR African descent OR African ancestry OR Africans |
| **S2 - EXPOSURE** |
| Traditional diet OR Diet OR Healthy dietary patterns OR Healthy diet OR Vegetarian diet OR Vegetarian OR Vegetarianism OR Vegan diet OR Vegan OR Plant based diet OR Mediterranean diet OR Dietary approaches to stop hypertension OR DASH diet OR dietary patterns OR eating behaviour OR nutrition |
| **S3 - COMPARATOR** |
| Unhealthy dietary patterns OR Westernized diet OR Western diet OR Animal based diet OR Fast foods |
| **S4 - HEALTH OUTCOMES** |
| Cardiovascular disease OR Metabolic syndrome OR Hypertension OR Diabetes mellitus OR Insulin resistance OR Hyperglycemia OR Dysglycaemia OR Prediabetes OR Dyslipidaemia OR Hypercholesterolemia OR Hypertriglyceridemia OR Obesity OR Overweight |
| **S5** |
| S1 AND S2 AND S3 AND S4 |
| **FILTERS AND/OR LIMITS APPLIED** |
| Limiters – Published Date: 1990/01/01-2021/12/31; Narrow by Subject Age: all adult:19+ years |
| **African Journals Online (AJOL)** |
| "Traditional diet" OR "Healthy dietary patterns" OR "Healthy diet" OR "Vegetarian diet" OR "Vegan diet" OR "Plant based diet" OR "Plant foods" OR "fruit and vegetable consumption" OR "Mediterranean diet" OR "DASH" |
| “Cardiovascular disease” OR “Metabolic syndrome” OR “Hypertension” OR “Diabetes mellitus” OR “Insulin resistance” OR “Hyperglycemia” OR “Dysglycaemia” OR “Prediabetes” OR “Dyslipidaemia” OR “Hypercholesterolemia” OR “Hypertriglyceridemia” OR “Obesity” OR “Overweight” |
| “plant-based diet” OR “cardiovascular disease risk” |
| "Traditional diet" OR "Healthy dietary patterns" OR "Healthy diet" OR "Vegetarian diet" OR "Vegan diet" OR "Plant based diet" OR "Plant foods" OR "fruit and vegetable consumption" OR "Mediterranean diet" OR "DASH" NOT animal |

| **ISI Web of Science** | |
| --- | --- |
| #1 - POPULATION | CU=Africa* OR CU=Algeria OR CU=Angola OR CU=Benin OR CU=Botswana OR CU=Burkina Faso OR CU=Burundi OR CU=Cameroon OR CU=Cameroun OR CU=Cape Verde OR CU=Central African Republic OR CU=République Centre Afrique OR CU=RCA OR CU=CAR OR CU=Chad OR CU=chad OR CU=Comoros Islands OR CU=Comoros OR CU=Congo Or CU=Democratic Republic of Congo OR CU=DRC OR CU=République Démocratique du Congo OR CU=RDC OR CU=Djibouti OR CU=Egypt OR CU=Equatorial Guinea OR CU=Eritrea OR CU=Ethiopia OR CU=Gabon OR CU=Gambia OR CU=Ghana OR CU=Guinea OR CU=Guinea Bissau OR CU=Ivory Coast OR CU=Cote dIvoire OR CU=Kenya OR CU=Lesotho OR CU=Liberia OR CU=Libya OR CU=Madagascar OR CU=Malawi OR CU=Mali OR CU=Mauritania OR CU=Mauritius OR CU=Mayotte OR CU=Morocco OR CU=Mozambique OR CU=Namibia OR CU=Niger OR CU=Nigeria OR CU=Principe OR CU=Sao Tome OR CU=Sao Tome & Principe OR CU=Rwanda OR CU=Senegal OR CU=Seychelles OR CU=Sierra Leone OR CU=Somalia OR CU=Somali Land OR CU=South Africa* OR CU=South Sudan OR CU=Sudan OR CU=Swaziland OR CU=Tanzania OR CU=Togo OR CU=Tunisia OR CU=Uganda OR CU=Western Sahara* OR CU=Zambia OR CU=Zimbabwe OR CU=Central Africa* OR CU=West Africa* OR CU=Western Africa* OR CU=East Africa* OR CU=Eastern Africa* OR CU=North Africa* OR CU=Northern Africa* OR CU=Southern Africa* OR CU=sub Saharan Africa* OR CU=sub-Saharan Africa* OR CU=Africa South of Sahara* OR TS=African descent OR TS=African ancestry OR TS=Africans OR TS=African Europeans* OR TS=African/Europeans* OR CU=America* OR TS=African Asians* OR TS=African/Asians* OR TS=African Americans* OR TS=African/Americans* OR TS=African migrants OR CU=Southern African Development Community OR CU=SADC OR TS=African Caribbeans* OR TS=African/Caribbeans |
| #2 - EXPOSURE | TS=Diet OR TS=Healthy dietary patterns OR TS=Healthy diet OR TS=Vegetarian diet OR TS=Vegetarian OR TS=Vegetarianism OR TS=Vegan diet OR TS=Vegan OR TS=Plant-based diet OR TS=Plant based diet OR TS=Plant based OR TS=Mediterranean diet OR TS=Dietary approaches to stop hypertension OR TS=DASH diet OR TS=dietary patterns OR TS=Eating behavior |
| #3 – COMPARATOR | TS=Unhealthy dietary patterns OR TS=Westernized diet OR TS=Western diet OR TS=Animal-based diet OR TS=Animal based diet |
| #4 – EXPOSURE HEALTH OUTCOMES | TS=Cardiometabolic health OR TS=Lower risk of cardiovascular disease OR TS=Reduced blood pressure OR TS=Glycemic control OR TS=Reduce insulin resistance OR TS=Healthy lipid profile OR TS=Health body weight OR TS=Lower body mass index |
| #5 – COMPARATOR OUTCOMES | TS=Cardiovascular disease OR TS=Cardiometabolic disease OR TS=Cardiometabolic risk OR TS=Cardiometabolic risk profile OR TS=Cardiovascular disease risk factors OR TS=Metabolic syndrome OR TS=Hypertension OR TS=Raised blood pressure OR TS=High blood pressure OR TS=Diabetes mellitus OR TS=Type 2 diabetes mellitus OR TS=Insulin resistance OR TS=Hyperglycemia OR TS=Dyslipidemia OR TS=Hypercholesterolemia OR TS=Hypertriglyceridemia OR TS=Obesity OR TS=Abdominal obesity OR TS=Adiposity OR TS=Body mass index OR TS=Overweight |
| #6 | #1 AND #2 AND #3 AND #4 AND #5 |
