# Supplementary material for: Assessment of the association between plant-based dietary exposures and cardiovascular disease risk profile in sub-Saharan Africa: a systematic review
Source: BMC Public Health. 2022 Feb 19;22:361. doi: 10.1186/s12889-022-12724-w (PMC8858494; doi:10.1186/s12889-022-12724-w)
Supplement: Supplementary file 4 — Additional file 4: Table S2. Quality assessment of observational studies using the NHLBI tools. Table S3. Plant-based dietary exposures identified across studies conducted in SSA between 2002 and 2020 [file 12889_2022_12724_MOESM4_ESM.docx]

**Additional file 4**

**Table S2. Quality assessment of observational studies using the NHLBI tools**

| **Year**  **[ref]** | **2020**  **[52]** | **2020**  **[46]** | **2019**  **[53]** | **2019**  **[44]** | **2018**  **[42]** | **2018**  **[47]** | **2018**  **[41]** | **2018**  **[51]** | **2018**  **[48]** | **2017**  **[49]** | **2017**  **[43]** | **2017**  **[40]** | **2016**  **[39]** | **2010**  **[50]** | **2002**  **[45]** |
| --- | --- | --- | --- | --- | --- | --- | --- | --- | --- | --- | --- | --- | --- | --- | --- |
| **Was the research question or objective in this paper clearly stated?** | | | | | | | | | | | | | | | |
| **Q1** | Y | Y | Y | Y | Y | Y | Y | Y | Y | Y | Y | Y | Y | Y | Y |
| **Was the study population clearly specified and defined?** | | | | | | | | | | | | | | | |
| **Q2** | Y | Y | Y | Y | Y | Y | Y | Y | Y | Y | Y | Y | Y | Y | Y |
| **Was the participation rate of eligible persons at least 50%?**  ****Did the authors include a sample size justification?** | | | | | | | | | | | | | | | |
| **Q3** | Y | Y | Y | Y | CD | NR | N | NR | Y | NR | Y | NR | Y | NR | Y |
| **Were all the subjects selected or recruited from the same or similar populations (including the same time period)? Were inclusion and exclusion criteria for being in the study prespecified and applied uniformly to all participants? **Were controls selected or recruited from the same or similar population that gave rise to the cases (including the same timeframe)?** | | | | | | | | | | | | | | | |
| **Q4** | Y | Y | Y | Y | Y | N | Y | Y | Y | Y | Y | Y | Y | Y | Y |
| **Was a sample size justification, power description, or variance and effect estimates provided? **Were the definitions, inclusion and exclusion criteria, algorithms or processes used to identify or select cases and controls valid, reliable, and implemented consistently across all study participants?** | | | | | | | | | | | | | | | |
| **Q5** | Y | Y | Y | Y | Y | Y | N | Y | Y | Y | N | N | Y | Y | CD |
| **For the analyses in this paper, were the exposure(s) of interest measured prior to the outcome(s) being measured?**  ****Were the cases clearly defined and differentiated from controls?** | | | | | | | | | | | | | | | |
| **Q6** | N | N | N | N | N | N | N | N | N | Y | N | N | Y | N | N |
| **Was the timeframe sufficient so that one could reasonably expect to see an association between exposure and outcome if it existed? **If less than 100 percent of eligible cases and/or controls were selected for the study, were the cases and/or controls randomly selected from those eligible?** | | | | | | | | | | | | | | | |
| **Q7** | N | N | N | N | N | N | N | N | N | Y | N | N | CD | N | N |
| **For exposures that can vary in amount or level, did the study examine different levels of the exposure as related to the outcome (e.g., categories of exposure, or exposure measured as continuous variable)? **Was there use of concurrent controls?** | | | | | | | | | | | | | | | |
| **Q8** | Y | Y | Y | Y | Y | Y | Y | Y | Y | Y | Y | Y | N | Y | NR |
| **Were the exposure measures (independent variables) clearly defined, valid, reliable, and implemented consistently across all study participants?**  ****Were the investigators able to confirm that the exposure/risk occurred prior to the development of the condition or event that defined a participant as a case?** | | | | | | | | | | | | | | | |
| **Q9** | Y | Y | Y | Y | Y | Y | Y | Y | Y | Y | Y | N | Y | Y | Y |
| **Was the exposure(s) assessed more than once over time?**  ****Were the measures of exposure/risk clearly defined, valid, reliable, and implemented consistently (including the same time period) across all study participants?** | | | | | | | | | | | | | | | |
| **Q10** | N | N | N | N | N | N | N | N | N | Y | N | N | Y | N | N |
| **Were the outcome measures (dependent variables) clearly defined, valid, reliable, and implemented consistently across all study participants?**  ****Were the assessors of exposure/risk blinded to the case or control status of participants?** | | | | | | | | | | | | | | | |
| **Q11** | Y | Y | Y | Y | Y | Y | Y | Y | Y | Y | Y | Y | NR | Y | Y |
| **Were the outcome assessors blinded to the exposure status of participants?**  ****Were key potential confounding variables measured and adjusted statistically in the analyses? If matching was used, did the investigators account for matching during study analysis?** | | | | | | | | | | | | | | | |
| **Q12** | N | N | N | N | N | N | N | N | N | N | N | N | Y | N | N |
| **Was loss to follow-up after baseline 20% or less?** | | | | | | | | | | | | | | | |
| **Q13** | NA | NA | NA | NA | NA | NA | NA | NA | NA | NR | NA | NA | - | NA | NA |
| **Were key potential confounding variables measured and adjusted statistically for their impact on the relationship between exposure(s) and outcome(s)?** | | | | | | | | | | | | | | | |
| **Q14** | Y | Y | Y | N | Y | Y | NR | NR | Y | Y | Y | NR | - | Y | Y |
| **Score** | **9/14** | **9/14** | **9/14** | **8/14** | **8/14** | **7/14** | **6/14** | **7/14** | **9/14** | **11/14** | **8/14** | **5/14** | **9/12** | **8/14** | **7/14** |
| **Quality** | **Fair** | **Fair** | **Fair** | **Fair** | **Fair** | **Fair** | **Fair** | **Fair** | **Fair** | **Fair** | **Fair** | **Poor** | **Fair** | **Fair** | **Fair** |

** - Criteria for NHLBI Quality Assessment of Case-Control Studies

Y – Yes; N – No; CD – Cannot determine; NR – Not reported; NA – Not applicable

**Table S3. Plant-based dietary exposures identified across studies conducted in Africa between 2002 and 2020**

| Plant-based dietary exposures | Dietary exposure | Health outcome | Reported measures of association between exposure and outcome | Significant association between exposure and outcome | Country name(s), [reference] |
| --- | --- | --- | --- | --- | --- |
| Consumption of plant foods | Fruit and vegetables | Overweight/obesity | Unadjusted and adjusted ORs with 95% CIs | No | Ethiopia [53] |
|  | Fruit and vegetables | Hypertension | Unadjusted and adjusted ORs with 95% CIs | Yes | Zambia [42] |
|  | Fruit and vegetables  Salads | Overweight/obesity | Mean and standard deviations with p-values | No | South Africa [51] |
|  | Fruit and vegetables | Overweight/obesity | Proportions with p-values | Yes | South Africa [40] |
|  | Cassava leaves  Dried red beans  Solo  Bitekuteku | MetS | Unadjusted and adjusted ORs with 95% CIs | Yes | DRC [50] |
|  | Green vegetables  Coconut milk | Obesity  Dyslipidaemia | β regression coefficients with p-values | Yes | Tanzania [45] |
|  | Fruit and vegetables | Hypertension | Mean with p-values | Yes | Kenya [44] |
|  | Fruit and vegetables | T2D | Proportions with p-values | No | South Africa [46] |
| Dietary and nutrient patterns characterized using principal component analysis (PCA) | Mixed diet pattern | Overweight/obesity | Proportions and ORs with 95% CIs | No | Nigeria, Tanzania,  South Africa, and Uganda [47] |
|  | Roots, tubers, and plantain | T2D | Unadjusted and adjusted ORs with 95% CIs | Yes | Ghana [48] |
|  | Healthy pattern  Complex carbohydrate pattern | Hypertension | ARRs with 95% CIs | Yes | Tanzania [43] |
|  | Fruit and vegetable dietary pattern  Fish and nuts dietary pattern  Botswana Traditional foods dietary pattern | Overweight/obesity | Unadjusted and adjusted RRs with 95% CIs | Yes | Botswana [52] |
|  | Plant driven nutrient patterns | T2D | β regression coefficients with 95% CIs and p-values | Yes | South Africa [49] |
| Indicator food categories | Fruit and vegetables  White bread and starches  Brown bread, legumes, high fibre cereals | Overweight/obesity | Mean and standard deviations or median and interquartile range with p-values | No | South Africa [41] |
| Dietary index | mAHEI  Daily fruit and vegetable intake | Acute stroke | Prevalence proportions, ORs and Population attributable risk with 99% CIs | No | Mozambique, Nigeria,  South Africa, Sudan, and Uganda [39] |
